# Bi-directional gene set enrichment and canonical correlation analysis identify key diet-sensitive pathways and biomarkers of metabolic syndrome

**DOI:** 10.1186/1471-2105-11-499

**Published:** 2010-10-07

**Authors:** Melissa J Morine, Jolene McMonagle, Sinead Toomey, Clare M Reynolds, Aidan P Moloney, Isobel C Gormley, Peadar Ó Gaora, Helen M Roche

**Affiliations:** 1 Nutrigenomics Research Group, School of Public Health, UCD Conway Institute of Biomolecular & Biomedical Research, University College Dublin, Belfield, Dublin 4, Ireland; 2 Teagasc, Animal and Grassland Research and Innovation Centre, Grange, Dunsany, County Meath, Ireland; 3 School of Mathematical Sciences, University College Dublin, Belfield, Dublin 4, Ireland; 4 UCD School of Medicine and Medical Sciences, UCD Conway Institute of Biomolecular & Biomedical Research, University College Dublin, Belfield, Dublin 4, Ireland

## Abstract

**Background:**

Currently, a number of bioinformatics methods are available to generate appropriate lists of genes from a microarray experiment. While these lists represent an accurate primary analysis of the data, fewer options exist to contextualise those lists. The development and validation of such methods is crucial to the wider application of microarray technology in the clinical setting. Two key challenges in clinical bioinformatics involve appropriate statistical modelling of dynamic transcriptomic changes, and extraction of clinically relevant meaning from very large datasets.

**Results:**

Here, we apply an approach to gene set enrichment analysis that allows for detection of bi-directional enrichment within a gene set. Furthermore, we apply canonical correlation analysis and Fisher's exact test, using plasma marker data with known clinical relevance to aid identification of the most important gene and pathway changes in our transcriptomic dataset. After a 28-day dietary intervention with high-CLA beef, a range of plasma markers indicated a marked improvement in the metabolic health of genetically obese mice. Tissue transcriptomic profiles indicated that the effects were most dramatic in liver (1270 genes significantly changed; p < 0.05), followed by muscle (601 genes) and adipose (16 genes). Results from modified GSEA showed that the high-CLA beef diet affected diverse biological processes across the three tissues, and that the majority of pathway changes reached significance only with the bi-directional test. Combining the liver tissue microarray results with plasma marker data revealed 110 CLA-sensitive genes showing strong canonical correlation with one or more plasma markers of metabolic health, and 9 significantly overrepresented pathways among this set; each of these pathways was also significantly changed by the high-CLA diet. Closer inspection of two of these pathways - selenoamino acid metabolism and steroid biosynthesis - illustrated clear diet-sensitive changes in constituent genes, as well as strong correlations between gene expression and plasma markers of metabolic syndrome independent of the dietary effect.

**Conclusion:**

Bi-directional gene set enrichment analysis more accurately reflects dynamic regulatory behaviour in biochemical pathways, and as such highlighted biologically relevant changes that were not detected using a traditional approach. In such cases where transcriptomic response to treatment is exceptionally large, canonical correlation analysis in conjunction with Fisher's exact test highlights the subset of pathways showing strongest correlation with the clinical markers of interest. In this case, we have identified selenoamino acid metabolism and steroid biosynthesis as key pathways mediating the observed relationship between metabolic health and high-CLA beef. These results indicate that this type of analysis has the potential to generate novel transcriptome-based biomarkers of disease.

## Background

The metabolic syndrome (MetS) describes a combination of metabolic abnormalities that increase risk of diabetes and cardiovascular disease. Although diet is not implicated as a risk factor, the onset of the MetS is at least partly triggered by energy dense, high-fat diets that promote obesity and insulin resistance [1]. Nutritional genomics strives to understand molecular-level metabolic effects of dietary components, and to develop sensitive tools to analyze these effects. This has proven to be a formidable challenge, as many nutrients have ubiquitous metabolic effects that are both subtle and complex [2]. In the case of MetS, this is further complicated by the involvement of multiple organs, including adipose tissue, liver and skeletal muscle.

Traditional metabolic biomarkers, such as plasma glucose and triglycerides, have well-established associations with health [3-5], but do not reflect the vast complexity of inter-organ metabolic processes. High-throughput 'omics' technologies address this limitation by assessing multiple cellular processes simultaneously, although this magnitude of data can become limiting in attempts to summarize clinical relevance of an 'omic profile. Combined analysis of plasma markers and high-throughput data can provide a richer source of information relevant to metabolic health. The approach used here - canonical correlation analysis (CCA) - reveals global correlation patterns between gene expression and plasma markers. In contrast to typical ranking based on fold-change or statistical evidence of differential expression, these correlation patterns can be used to rank the 'importance' of diet-sensitive genes based on the degree of correlation with diagnostic markers.

Another novel approach used here is the identification of bi-directional enrichment in biochemical pathways - a concept that was developed by Saxena *et al. *[6] and implemented by Dinu *et al. *[7] but is still not routinely used, particularly in clinical studies. The generic procedure in gene set enrichment analysis (GSEA) involves defining gene sets (most commonly, KEGG biochemical pathways), and identifying coordinated regulation of these sets. However, simple up- or down-regulation of gene sets does not always capture the subtlety of pathway biology. For example, the glycolysis/gluconeogenesis pathway involves both glucose catabolism and anabolism. Given a biological perturbation, such as a dietary treatment, these two processes may be regulated in opposing directions. In such cases, whole-pathway single-direction regulation would not adequately reflect observed patterns in expression changes. The modification to the GSEA algorithm presented here provides a computational adjustment that allows for detection of these bi-directional changes, providing more precise understanding of transcriptome regulation.

As a proof of principal, this study focused on *cis-*9, *trans*-11 conjugated linoleic acid (CLA), a fatty acid isomer with anti-diabetic and anti-inflammatory properties [8,9]. Given the complex multi-tissue basis of the pathophysiology of MetS, our aim was to determine the metabolic and inter-organ transcriptomic signature of the three insulin-sensitive tissues (adipose, skeletal muscle and liver) of obese and diabetic mice fed a diet high in beef-derived *cis-*9, *trans*-11 CLA.

## Methods

### Animal experimentation

The animal feeding experiment was conducted at the BioResources Unit, Trinity College Dublin (TCD) Ireland according to European Union (EU) animal research welfare protocol, with approval for experimentation granted by the Department of Health and Children in Ireland (License number B100/3041). Fourteen, 4-week-old male, *ob/ob *(C57BL/6J) mice were purchased from Harlan, UK. The mice were acclimatised for 7 days during which time they received a purified control diet, before being assigned to one of two treatment groups for a 28-day period. During the intervention period, the animals were exposed to 12 hrs light/12 hrs dark cycles, maintained at a constant temperature of 22°C.

### Dietary composition and preparation of the animal feeds

Diets were produced by Special Diets Service, Essex, UK and were received as 1 kg vacuum packed, heat sealed plastic bags. Low-CLA and high-CLA beef (0.53 and 2.65 w/w% of *c*9,*t*11 CLA, respectively) were provided by Teagasc (Grange Research Centre, Dunsany, Co. Meath). Test diet blends were prepared by mixing the beef component at a 36% inclusion rate to equal portions of wheat feed and maize (corn) feed. Final feeds were prepared by mixing 100 ml warm water with 100 g test diet blend. Dietary food intake was measured daily and the animals received freshly prepared food each day.

### Blood sample and tissue collection and handling protocol

The mice were sacrificed at day 28 of the dietary intervention period. Food was removed from the cages at 6:00 pm and the animals were sacrificed the following morning between 8:00 am - 10:00 am, in the fasted state. The animals were euthanized using Carbon Dioxide (CO_2_) and cardiac puncture was performed to draw blood samples. Blood was transferred to a cooled sodium citrate blood vacutainer tube (BD Vacutainer, Dublin, Ireland) and centrifuged at 1500 rpm for 15 mins at 4°C, plasma was harvested, aliquoted and stored (-70°C). Tissue samples for gene expression analysis were harvested, immediately immersed in 0.5 ml RNALater (Ambion, AMS Techonology) and stored (-70°C). RNA was later extracted using a Qiagen RNeasy extraction kit, and outsourced to ServiceXS http://www.servicexs.com for hybridization to Affymetrix arrays, custom designed by the European Nutrigenomics Organization containing 15313 probesets. This platform is designated 'nugomm1a520177', and we used the 'entrezg' version 12.1.0 annotation from the MBNI custom cdf database http://brainarray.mbni.med.umich.edu/Brainarray/Database/CustomCDF/genomic_curated_CDF.asp, which reflects the latest remapping of Affymetrix probes based on current data in the NCBI database [10]. The complete array data are available at the GEO database under accession GSE23337.

### Determination & statistical analysis of plasma markers of metabolic syndrome

Plasma glucose concentrations were analysed using an endpoint enzymatic glucose oxidase, peroxidase, chromogen sequence, colorimetric assay (Biomérieux, France). A multiplex ELISA assay kit manufactured by Linco Research (Missouri, USA) was used to simultaneously quantify insulin, TNFα, MCP-1, resistin, and PAI-1 concentrations from mouse serum samples, while IL-6 and adiponectin were measured using ELISA kits from BioSources International (California, USA) and R&D Systems (Minnesota, USA), respectively. Plasma triglycerides (TAG) and cholesterol levels were measured using enzymatic-based assays from Randox Laboratories (Co. Antrim, UK), while plasma non-esterified fatty acids (NEFA) were quantified using a Randox NEFA kit. Insulin resistance (as defined by the homeostasis model assessment insulin resistance index; HOMA_IR_) was calculated as [fasting glucose (mg/dl) multiplied by fasting insulin (μU/ml)] divided by 22.5 [11]. Significance of plasma marker level variation between groups was determined using ANOVA in conjunction with Tukey's honest significant differences test, which corrects for experiment-wise error rate.

### Processing of microarray data, and single gene statistical analysis

Raw microarray data were first assessed for quality using a set of standard QC tests, including array intensity distribution, positive and negative border element distribution, GAPDH and ß-actin 3':5' ratios, centre of intensity and array-array correlation check. All QC tests were implemented in the R programming language [12], using the affyQCReport library [13]. After quality assessment, all intensity values were background corrected and normalized (within each tissue group) using the GCRMA-slow method (which uses a slower and more exact optimization algorithm) [14]. Probesets were filtered to remove genes with low or null expression, using a filter wherein probesets showing an intensity score less than 3 on more than 50% of the arrays were removed. Filtered adipose, liver and skeletal muscle datasets comprised 8575, 7781 and 8093 probesets, respectively. Single gene analysis was carried out using the LIMMA library [15], wherein linear models were fitted to each probeset on the array, to determine statistical significance of the effect of the high-CLA beef diet. Empirical Bayes statistics were generated using the eBayes() function, and resultant p-values were adjusted for multiple testing, using the Benjamini & Hochberg method [16].

### Gene set enrichment analysis

A script in R was written to carry out gene set enrichment analysis on each tissue dataset, adapting the statistical code provided in the GSEAlm library in R (Additional file 1) [17]. In addition to the typical single-direction enrichment, an additional test was included where absolute values of t-statistics were used, to detect bi-directional enrichment. T-statistics were extracted from linear models, which were fitted to each gene in a given gene set (*i.e*., KEGG pathway) - using 'diet group' as the predictor variable and 'expression level' as the response. These t-statistics (absolute values of t-statistics for bi-directional enrichment analysis) were then summed, and normalized for the number of genes in the gene set. Diet group labels were then randomized, as in a typical permutation test, and gene-set t-statistics were re-calculated using these randomized groupings. This permutation step was repeated 1000 times, and p-values were calculated by determining the proportion of permutation t-statistics that were closer to zero than the 'true' t-statistic. For instance, a p-value of 0.05 would be recorded if the original t-statistic were greater than more than 95% of the permutation t-statistics. These p-values were corrected using the Benjamini & Hochberg method [16]. R scripts were written to produce summary plots of the results, and also to import KEGG pathway data, integrate microarray results, and export the annotated pathway to Cytoscape http://www.cytoscape.org for visualisation.

### Regularized canonical correlation analysis

To determine canonical correlations between metabolic and transcriptomic data, gene expression and metabolic marker values were centered to 0 and scaled to have variance 1 (*i.e*, z-score normalized) within each diet group, to reveal the null correlations between gene expression and metabolic markers, irrespective of dietary treatment. To make CCA results more easily comparable to GSEA results, we used the subset of genes in our expression dataset with annotation to a KEGG pathway. The 'mixOmics' library of functions in R was used to carry out the analysis [18]. Specifically, the *rcc *function was used to define the canonical correlations and the canonical variates, *estim.regul *for estimation of regularization parameters and the *network *function to produce the initial network of interactions. An additional script was written to output the R network to Cytoscape for visualization. Taking the group of genes with a correlation score of at least .65 (using 'threshold' argument of the *network *function in the mixOmics library; for further information on this association measure see [19]) with at least one plasma marker, Fisher's exact test was performed to define pathways that were significantly overrepresented among MetS-associated genes [20].

## Results

### High-CLA beef diet improved insulin sensitivity, lipoprotein profile and inflammatory status

**Figure 1:**
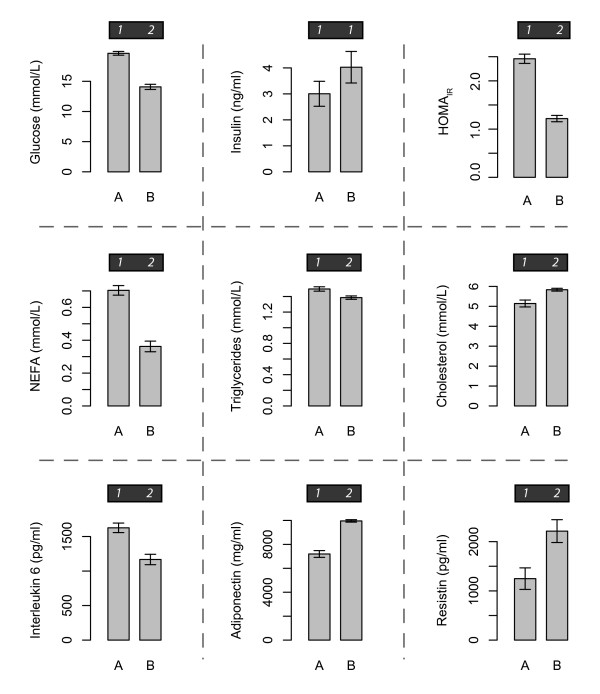
**Changes in plasma markers of MetS, in response to dietary fat modification: A = control LA-enriched diet; B = high-*c*9,*t*11 CLA beef enriched diet**. Error bars represent standard error of the mean. Numbers above plots indicate statistically distinct groups (*i.e*., different numbers represent significant (α = 0.05) differences between diet groups).

Feeding a diet enriched with natural beef derived *cis-*9, *trans-*11-CLA diet significantly reduced fasting plasma glucose (p = 5.6e-06) concentrations compared to control linoleic acid (LA)-enriched diet (Diet A) (Figure 1). While plasma insulin concentrations were not significantly different between diets, insulin resistance (HOMA_IR_) was significantly improved after the high-CLA beef diet, compared to the control diet (*p *= 5.1e-06).

There was a marked improvement in plasma TAG and NEFA concentrations following the high-CLA beef diet (Diet B), compared to the control diet (*p *= 0.023 and 5.56e-05). In contrast, total serum cholesterol was significantly increased in the high-CLA beef diet. Total serum adiponectin, an adipokine that promotes metabolic health, was significantly increased after feeding the high-CLA beef diet (p = 8.4e-05), while serum IL-6 concentrations were significantly reduced by the high-CLA beef diet, compared with the control group (p = 0.008). Counterintuitively, serum resistin concentrations were significantly higher following the high-CLA diet (p = 0.019). Plasma TNFα, PAI-1 and MCP-1 were not significantly different between the diets (data not shown). Overall, these plasma marker results suggest that the high-CLA beef diet induced changes in glucose homeostasis, lipid metabolism and systemic inflammation reflective of an improvement in metabolic health.

### Single-gene transcriptomic analysis reveals a spectrum of regulatory responses across insulin sensitive tissues

**Figure 2:**
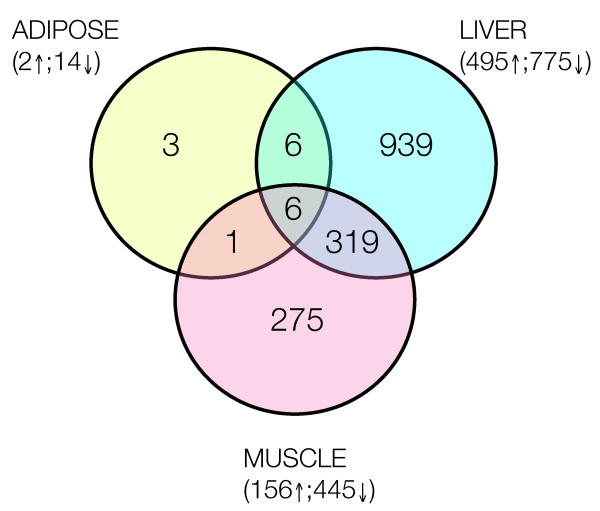
**Summary of single-gene changes in expression in liver, muscle and adipose tissue, in response to feeding obese, diabetic *ob/ob *mice a CLA-enriched beef diet that improved metabolic markers associated with diabetes**.

Figure 2 summarizes the results of single-gene analysis in adipose, skeletal muscle and liver tissue from mice fed the control diet (Diet A) or high-CLA beef diet (Diet B). The strongest effects are clearly manifest in liver tissue, with expression changes observed in 1270 genes (p < 0.05). Of these, 495 were up-regulated and 775 down-regulated, in response to the high-CLA diet. Skeletal muscle displayed more modest changes, with 156 genes up-regulated and 445 down-regulated (601 total). In adipose tissue, only 16 genes were found to be significantly regulated by diet - 2 up-regulated and 14 down-regulated. Overall, there was very little global overlap in observed transcriptomic changes; only 6 genes were significantly regulated by diet across all tissues. This finding was somewhat surprising; despite the divergent biological roles of these tissues, all three tissues share core functions associated with nutrient handling. Such tissue-specific effects of the high-CLA diet highlight the value of multi-tissue perspective in nutritional intervention studies of MetS.

### GSEA indicates diverse pathway changes in response to CLA-rich beef

**Figure 3:**
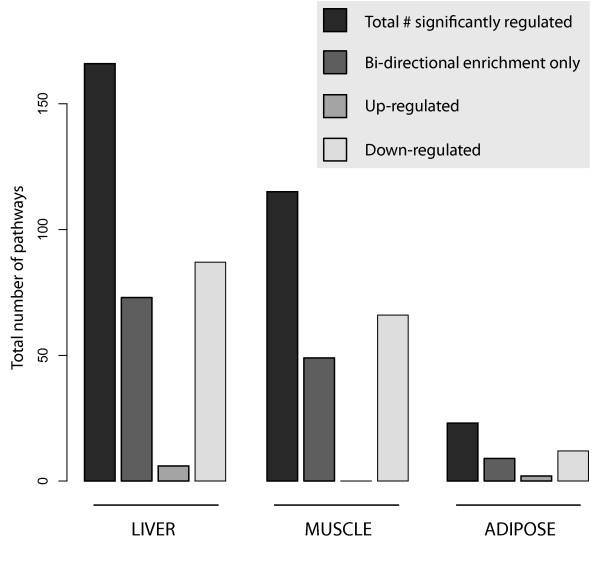
**Numbers of pathways affected by high-CLA diet**. The relative contribution of up-, down- and bi-directional regulation to total observed changes in KEGG pathways (α = 0.05) is illustrated.

**Figure 4:**
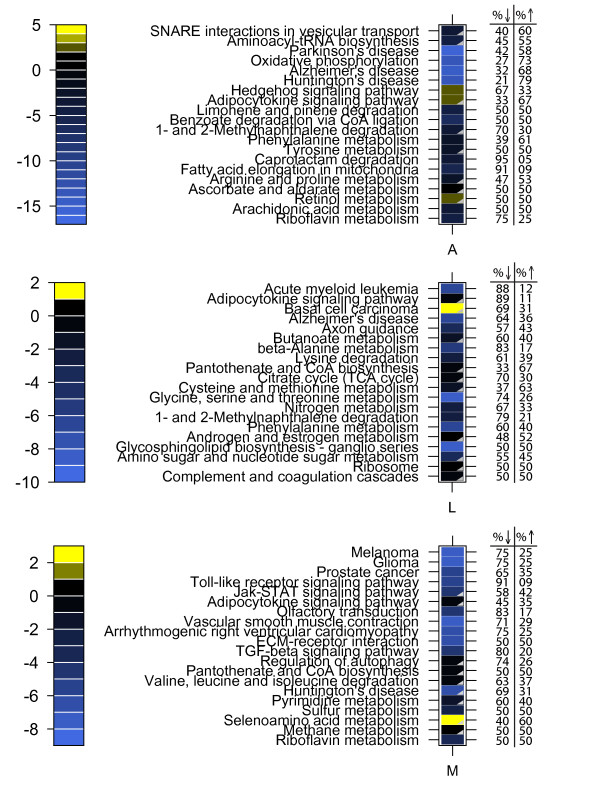
**Top 20 regulated KEGG pathways in adipose (A) liver (L) and muscle (M) in response to feeding obese, diabetic *ob/ob *mice a CLA-enriched beef diet that improved plasma markers associated with metabolic health**. Heatmap colours indicate pathway-level t-statistics. Yellow: up-regulated; blue: down-regulated. Grey triangles indicate pathways wherein bidirectional, but not single direction enrichment, reached significance (α = 0.05; adjusted for multiple testing). Values indicate percentage of up-regulated and down-regulated genes among those with raw p-value < 0.05.

GSEA results showed 166 significantly changed pathways in liver, 115 in skeletal muscle, and 23 in adipose tissue (Additional file 2; Additional file 3; Figure 3). Altered pathways - particularly in liver and muscle - spanned a range of biological processes, including metabolism, signalling and immune response. Figure 4 shows the top 20 significantly regulated pathways (ordered by proportion of constituent genes significantly regulated) in adipose, liver and muscle. In adipose tissue, top regulated pathways are primarily involved in cell signalling, and metabolism of energy, fat, amino acids, xenobiotics, secondary metabolites and vitamins. Top pathway changes in liver are in the categories of cell signalling, immune response, translation, and metabolism of carbohydrates, energy, amino acids, hormones, glycans and xenobiotics. Muscle tissue pathway changes showed strongest regulation of signalling pathways, cancer, cell transport, and metabolism of nucleotides, amino acids, energy and vitamins.

### GSEA indicates that nearly half of the expression changes in KEGG pathways are bi-directional

Figure 3 summarizes the results from GSEA in terms of the total number of significantly regulated pathways in each tissue, as well as the fraction that were significant only in the bi-directional test. In this figure, 'up-regulated' and 'down-regulated' pathways represent results obtained with a typical single-direction GSEA. Pathways in the second 'bi-directional enrichment only' column were significantly enriched using the bi-directional test, but not with the traditional GSEA method. This figure reveals considerable prevalence of this type of pathway regulation, and that potentially important biological results may be missed with single-direction enrichment tests.

### Regularized canonical correlation analysis identifies diet-sensitive genes and pathways showing strong association with metabolic health

Canonical correlation analysis (CCA) is a multivariate statistical technique, used to infer correlation patterns between two sets of variables measured on the same observations [21]. As standard CCA algorithms cannot be directly applied to high-throughput datasets (due to multicollinearity in very large correlation matrices), modified CCA algorithms - such as sparse [22] or regularized [23] CCA - have been developed to specifically deal with high dimensional datasets. Such regularized forms of CCA have been applied in joint analyses of SNP/transcriptomic [22,24] and transcriptomic/proteomic [25] datasets to reveal novel disease biomarkers and clarify inter-tissue molecular interactions.

**Figure 5:**
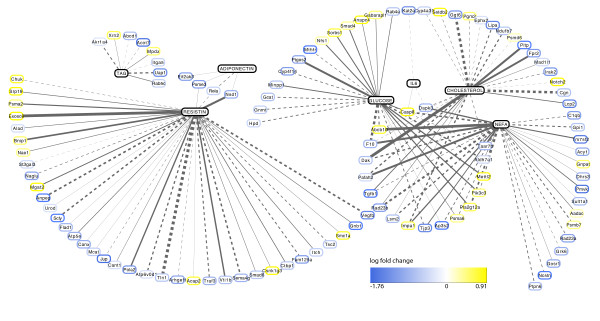
**CCA of hepatic gene expression profiles and phenotype markers of metabolic health**. Log fold-change of genes, in response to the high CLA diet, are indicated in heatmap colours, ranging from blue (down-regulated) to yellow (up-regulated); log fold change values are indicated in the legend; the width of connecting lines corresponds to the strength of correlation (dashed lines = negative correlations; solid lines = positive).

As the liver gene expression changes were the strongest across the three tissues, Figure 5 shows the results of regularized CCA between plasma markers and liver gene expression levels. To highlight only the strongest marker-gene associations, genes in this figure are the subset that display a correlation score (*i.e*., using the 'threshold' argument of the *network *function in the mixOmics library) greater than 0.65 with at least one of the plasma markers of metabolic health, and showed significant diet-sensitive expression in the single gene analysis. Width of connecting lines in this diagram represents the magnitude of multidimensional plasma marker-gene associations (calculated using the *network *function in the mixOmics library in R). The number of dimensions used in the calculation is chosen as the number that adequately describes associations in the dataset (as in principal component analysis; in this case, *n *dimensions = 6).

**Figure 6:**
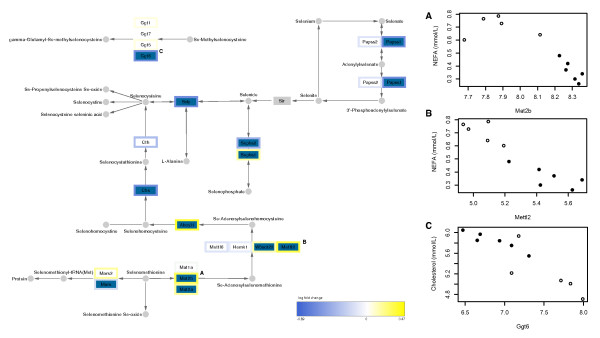
**High-CLA beef induced expression changes to selenoamino acid metabolism**. Genes indicated in dark blue showed significant change in expression given the high-CLA diet (raw p-value < 0.05). Genes indicated in white were not significantly changed or showed very low expression and were removed from the dataset, while those in grey were not tested on the microarray. Node border indicates log-fold change using heatmap colours, as described in the figure legend, and small, round nodes represent nutrients/metabolites. Scatterplots illustrate the strongest three gene-plasma markers correlations. In the scatterplots, open circles represent control diet-fed animals, and filled circles represent high-CLA beef-fed animals.

**Figure 7:**
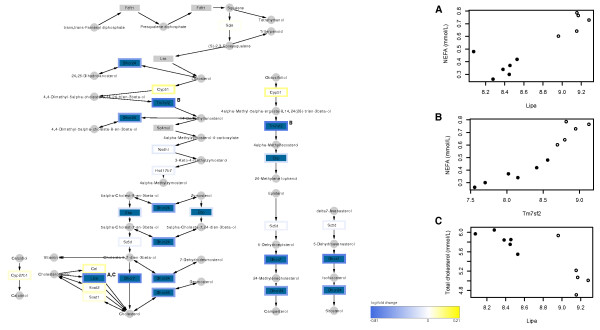
**High-CLA beef induced expression changes to steroid biosynthesis**. Genes indicated in dark blue showed significant change in expression given the high-CLA diet (raw p-value < 0.05). Genes indicated in white were not significantly changed or showed very low expression and were removed from the dataset, while those in grey were not tested on the microarray. Node border indicates log-fold change using heatmap colours, as described in the figure legend, and small, round nodes represent nutrients/metabolites. Scatterplots illustrate the strongest three gene-plasma markers correlations. In the scatterplots, open circles represent control diet-fed animals, and filled circles represent high-CLA beef-fed animals.

These results highlight CLA-sensitive genes that are correlated with multiple markers of metabolic health. For example, *f10, taar7b *and *abcb10 *show strong correlation with glucose, NEFA and cholesterol levels. *Casp8 *is strongly correlated with glucose, NEFA and IL-6, and *dapk3 *with NEFA, IL-6 and cholesterol. IL-6 and TAG show comparatively weaker gene-marker correlations than the other plasma markers. Fisher's exact test reveals pathways that are statistically overrepresented among these MetS associated genes - *i.e*., pathways showing stronger correlations with plasma markers than expected by chance (Table 1). Each of these pathways was also significantly changed in liver by the CLA diet (Table 2). Figures 6 and 7 illustrate the correlations between gene expression, diet and plasma markers of metabolic syndrome in the context of the selenoamino acid metabolism and steroid biosynthesis pathways. In each pathway, the three highest gene-plasma marker correlations are illustrated in scatterplots (Figure 6 and 7A-C).

**Table 1:** Fisher's exact test for statistically overrepresented pathways among CCA results.

Pathway	Odds Ratio	Expected Count	Actual Count	Pathway Size	p value
Selenoamino acid metabolism	3.240	3.694	8	17	0.018
Neuroactive ligand-receptor interaction	2.265	7.388	13	34	0.020
Histidine metabolism	2.915	3.911	8	18	0.026
Arginine and proline metabolism	2.363	6.084	11	28	0.026
Fc epsilon RI signaling pathway	2.192	6.953	12	32	0.030
Pancreatic cancer	1.961	9.343	15	43	0.032
Glycerolipid metabolism	2.523	4.780	9	22	0.033
Steroid biosynthesis	3.630	2.173	5	10	0.045
Heparan sulfate biosynthesis	3.630	2.173	5	10	0.045

Expected count represents the expected number of MetS associated genes in each pathway based on the overall number of MetS associated genes in the dataset, total number of genes tested, and size of the pathway.

**Table 2:** Subset of results from high-CLA beef diet GSEA (liver tissue) for pathways found to be related to MetS.

Pathway	bi-directional (p value)	up-regulation (p value)	down-regulation (p value)
Selenoamino acid metabolism	< 1e-8	0.956	0.499
Neuroactive ligand-receptor interaction	< 1e-8	0.592	0.892
Histidine metabolism	< 1e-8	1.000	0.331
Arginine and proline metabolism	< 1e-8	1.000	< 1e-8
Fc epsilon RI signaling pathway	0.003	1.000	0.003
Pancreatic cancer	0.003	1.000	0.119
Glycerolipid metabolism	0.003	1.000	0.018
Steroid biosynthesis	< 1e-8	1.000	< 1e-8
Heparan sulfate biosynthesis	0.062	1.000	0.006

## Discussion

### Bi-directional enrichment

As the field of transcriptomic methodology is still under development, it is important to recognize shortfalls in current methodology, and make improvements where possible. One such improvement presented here is in the identification of bi-directional enrichment in biochemical pathways. Although GSEA has added substantially to the interpretability of transcriptomic data, commonly used repositories for defining gene sets - such as the KEGG database - were not designed for such an application. As a result, in many KEGG pathways, single-direction enrichment is not biologically relevant or even feasible. A clear example of this concept is seen in signalling pathways, which often contain negative transcription regulation interactions or parallel sub-pathways under opposing regulatory control. Such cases of *de facto *bi-directional regulation may represent a substantial fraction of transcriptomic regulatory activities. Accordingly, our results show that among all significantly regulated pathways, nearly half of the observed changes were only significant with bi-directional, and not single-direction enrichment tests (Figure 3).

The prevalence of bi-directional regulation is further illustrated in Figure 4, wherein nearly half of observed changes in the top 20 regulated pathways reached significance with the bi-directional test only. Further, these results illustrate that - as expected - bi-directionally regulated pathways typically contain an even proportion of up-regulated and down-regulated genes. These findings suggest that bi-directional regulation contributes substantially to global pathway changes and that important pathway effects may be missed in using a traditional approach to GSEA.

### Canonical correlation analysis

A typical single-gene analysis of gene expression data produces a list of genes that are significantly regulated by a given treatment condition. This list can contain hundreds, or thousands of genes, thereby necessitating a criterion for ranking the relative importance of each gene in the list. Common ranking criteria, including fold-change or p-value have limited inherent biological relevance. There is no reason to assume that greater fold change indicates greater biological importance *per se*. Rather, these criteria are indicative of the reliability and reproducibility of the observed differences. The plasma markers measured in the present study, on the other hand, have clear biological and clinical relevance [26,4]. Canonical correlation analysis was applied here to hepatic expression data to assess correlation patterns between gene expression and plasma markers of MetS. Results from this analysis highlight a number of CLA-sensitive genes, such as *f10, casp8 *and *taar7b*, showing strong correlations with multiple phenotypic markers of metabolic health (Figure 5).

These CCA results - in conjunction with Fisher's exact test and GSEA results for CLA-sensitive pathways - reveal that selenoamino acid metabolism is correlated with the CLA dietary intervention as well as with plasma markers of metabolic syndrome (Figure 6). This pathway was significantly bi-directionally regulated by the high-CLA beef diet, but was not significantly regulated according to the single-direction test. The selenoamino acid metabolism pathway encompasses reactions involved in metabolism of the trace mineral selenium and selenium-bound amino acids, such as selenohomocysteine and selenomethionine. Selenoamino acids are incorporated into selenoproteins (*e.g*. glutathione peroxidase and thioredoxin) that have strong associations with health status; deficiency in the liver has been linked to liver cirrhosis and hepatomegaly in rats [27], and impaired immune response in mice [28]. Selenium is also well known for its anti-oxidant activities, and as such has been extensively studied in relation to cancer progression [reviewed by [29] and [30]]. Effects of selenium and selenoamino acids on cardiovascular health have also been reported; in rats, selenium deficient diets have been shown to increase hepatic triglyceride and VLDL secretion and fatty acid oxidation [31], and increase plasma cholesterol levels [32]. More recently, Sengupta *et al. *showed that targeted deletion of hepatic selenocysteine tRNA in mice resulted in elevated plasma cholesterol and increased expression of genes involved in cholesterol biosynthesis [33]. Future work should aim to assess variability in selenium content high-CLA beef, to determine if the observed alteration in selenium metabolism is a direct effect of CLA isomers or if it is due to variable selenium content. Although micro-nutrient data were not available in the present study, parallel proteomics work in our research group showed a significant reduction in hepatic selenium-binding protein 1 in mice fed a diet supplemented with pure *c*9,*t*11 CLA isomers (p = 0.013; unpublished data).

Our combined GSEA and CCA results also indicated significant correlation between the high-CLA diet, plasma markers of MetS, and the steroid biosynthesis pathway (Figure 7). This pathway has well-documented association with hepatic function and metabolic health; Buqué *et al. *recently reported significant over-expression of steroid biosynthesis genes in fatty liver of obese Zucker rats [34], and Woo *et al*., demonstrated that plasma hyperhomocysteinemia (a cardiovascular risk factor) induces hepatic cholesterol biosynthesis and subsequent lipid accumulation [35]. Interestingly, this study by Woo *et al. *also showed significant elevation of plasma cholesterol in response to induction of hepatic cholesterol biosynthesis, whereas the present study shows clear reduction of cholesterol biosynthetic gene expression in conjunction with elevated plasma cholesterol levels. This observed pattern could be explained by a feedback system between cholesterol biosynthesis and plasma cholesterol, as it has previously been shown that sterol starvation in HepG2 cells induces expression of *tm7sf2 *(a gene that encodes an early step enzyme in cholesterol biosynthesis) [36], and the finding that *tm7sf2 *expression is controlled by cellular sterol levels through the activity of the Sterol-responsive element-binding protein 2 (SREBP2) transcription factor [37]. The particularly strong positive correlation observed here between *tm7sf2 *expression and plasma NEFA levels (r = 0.94; even stronger than observed correlation between *tm7sf2 *and plasma cholesterol: r = -0.71) highlights this gene as a candidate for directed studies of high-CLA beef, hepatic function and plasma lipid profile.

## Conclusions

A key challenge in transcriptomics involves drawing simple biological conclusions from complex expression patterns; often, a microarray analysis focuses on a small subset of genes on the array, excluding hundreds or thousands for the sake of analytical simplicity. Results such as those presented here illustrate how CCA provides a means to define a clinically relevant objective criterion (*i.e*., plasma markers) by which to rank the relative importance of observed expression changes, and can be applied to provide biological context for large GSEA datasets. In using these methods jointly, we have highlighted pathways - including selenoamino acid metabolism and steroid biosynthesis - showing particularly strong relationships with both dietary treatment and metabolic health. As the clinical importance of these pathways would have been difficult to uncover without the aid of CCA, we propose that this methodology is useful in studies seeking to define treatment-sensitive diagnostic and prognostic biomarkers of disease.

## Authors' contributions

MJM designed and carried out the data analysis, and drafted the manuscript. JM, ST and CMR carried out the feeding trials, tissue harvesting and plasma marker measurements. APM assisted in production of high-CLA beef and study design. ICG contributed to statistical analyses, and PG to bioinformatic analyses and manuscript preparation. HMR conceived of the study, and participated in coordination of the work and preparation of the manuscript. All authors read and approved the final manuscript.

## Supplementary Material

Additional file 1 **bi-directional GSEA script**. R script used to carry out bi-directional gene set enrichment analysis. Adapted from *gsealmPerm *function in GSEAlm package [17]. Click here for file

Additional file 2 **Figure S1: Full GSEA results for high-CLA-diet effects in liver, muscle and adipose tissue**. Heatmap showing bi-directional GSEA results for KEGG pathways in liver, muscle and adipose tissue. Click here for file

Additional file 3 **Figure S1 legend**. Descriptive legend for figure S1. Click here for file
