# Supplementary figures and images for: Bi-directional gene set enrichment and canonical correlation analysis identify key diet-sensitive pathways and biomarkers of metabolic syndrome
Source: BMC Bioinformatics. 2010 Oct 7;11:499. doi: 10.1186/1471-2105-11-499 (PMC3098081; doi:10.1186/1471-2105-11-499)

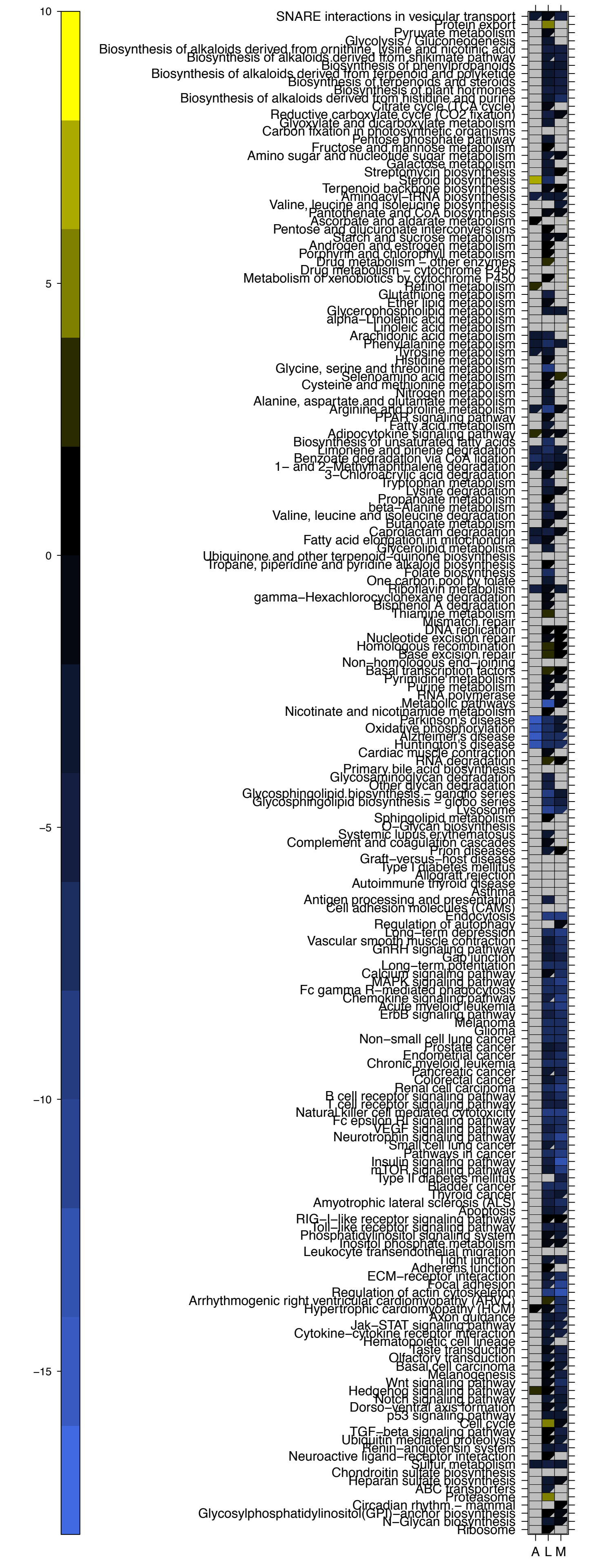

Supplement: Additional file 2 — Figure S1: Full GSEA results for high-CLA-diet effects in liver, muscle and adipose tissue. Heatmap showing bi-directional GSEA results for KEGG pathways in liver, muscle and adipose tissue. [file 1471-2105-11-499-S2.PDF]
