# Supplementary material for: Bi-directional gene set enrichment and canonical correlation analysis identify key diet-sensitive pathways and biomarkers of metabolic syndrome
Source: BMC Bioinformatics. 2010 Oct 7;11:499. doi: 10.1186/1471-2105-11-499 (PMC3098081; doi:10.1186/1471-2105-11-499)
Supplement: Additional file 3 — Figure S1 legend. Descriptive legend for figure S1. [file 1471-2105-11-499-S3.DOC]

Supplementary Figure S1. GSEA results for expression changes of KEGG pathways in adipose (A) liver (L) and muscle (M) in response to feeding obese, diabetic *ob/ob* mice a CLA-enriched beef diet that improved metabolic markers associated with diabetes. Heatmap colours indicate pathway-level t-statistics. Yellow: up-regulated; blue: down-regulated; grey: no significant change. Grey triangles indicate pathways wherein bidirectional, but not single direction enrichment, reached significance (α=0.05; adjusted for multiple testing).
